# Supplementary material for: Time-Averaged Hematuria as a Prognostic Indicator of Renal Outcome in Patients with IgA Nephropathy
Source: J Clin Med. 2022 Nov 16;11(22):6785. doi: 10.3390/jcm11226785 (PMC9694958; doi:10.3390/jcm11226785)
Supplement: Supplementary file 1 [file jcm-11-06785-s001.zip › jcm-2018802-supplementary.pdf]

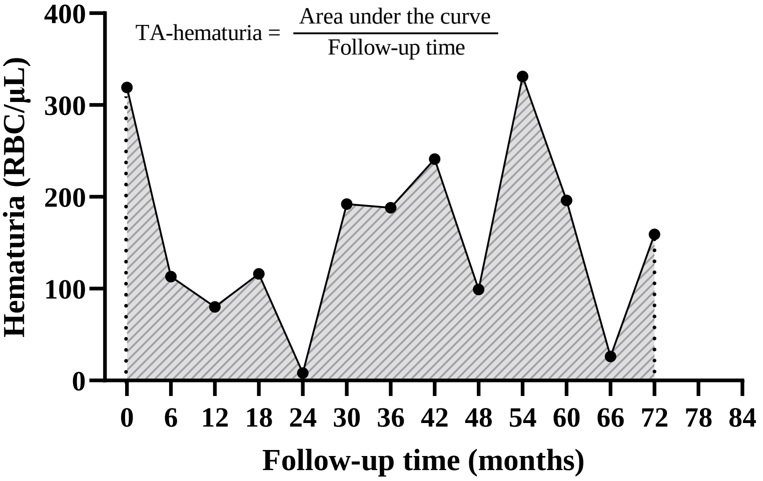

Supplementary Figure S1. Serial measurements of proteinuria during follow-up in one of the par-ticipants in this study and the definition of time-averaged hematuria (TA-hematuria).
